# Sediment exposure decreases diversity in the surface mucus layer microbiome of *Porites lobata* at Honoliʻi, Hawaiʻi

**DOI:** 10.3389/fmicb.2025.1626064

**Published:** 2025-07-28

**Authors:** Joseph W. P. Nakoa, John H. R. Burns, Makoa Pascoe, Manuela Cortes, Sofia B. Ferreira, Kailey H. Pascoe, Haunani H. Kane, Clifford A. Kapono

**Affiliations:** ^1^School of Life Sciences, Arizona State University, Tempe, AZ, United States; ^2^MEGA Lab, Hilo, HI, United States; ^3^Marine Science, Data Science, and Tropical Conservation Biology and Environmental Science, College of Natural and Health Sciences, University of Hawai’i at Hilo, Hilo, HI, United States; ^4^School of Ocean and Earth Science Technology, University of Hawaiʻi at Mānoa, Honolulu, HI, United States; ^5^School of Ocean Futures, Arizona State University, Tempe, AZ, United States

**Keywords:** coral microbiome, surface mucus layer, sedimentation stress, *Porites lobata*, microbial diversity, indicator taxa

## Abstract

**Introduction:**

Coral reefs are diverse marine ecosystems that provide essential ecological services, yet they are becoming increasingly degraded by anthropogenic stressors. Sediment deposition from land-based runoff can smother corals, reduce light availability, and alter the chemical and microbial composition of the water column. Prolonged sediment exposure disrupts coral-associated microbial communities, particularly within the surface mucus layer (SML), a physical barrier that mediates host–microbe interactions.

**Methods:**

We investigated shifts in the SML microbiome of *Porites lobata* corals in response to an acute sedimentation event at Honoliʻi, Hawaiʻi. Microbial community structure was characterized using 16S rRNA gene sequencing, at three time points, before, during, and after the sedimentation event, to identify changes in microbial composition and diversity.

**Results:**

Sedimentation caused a significant decline in microbial diversity and shifted community composition, with the most pronounced changes observed post-sedimentation. Indicator species analyses identified 206 bacterial taxa associated with specific sedimentation periods, including enrichment of Flavobacteriaceae during sedimentation and dominance of Endozoicimonaceae after sedimentation.

**Discussion:**

These findings demonstrate that sedimentation induces both immediate and delayed shifts in the SML microbiome, with potential implications for coral resilience. This study advances our understanding of how sedimentation affects coral-associated microbiomes and emphasizes the need to investigate the functional roles of microbial taxa involved in community transitions and recovery to inform conservation strategies.

## 1 Introduction

Coral reefs support approximately 25% of all marine species while covering less than 0.1% of the ocean floor (Knowlton et al., 2010). These ecosystems provide essential ecological and economic services, including fisheries, tourism, and coastal protection, with reef-related economic activities estimated to generate $38.5 billion annually (Moberg and Folke, 1999; Ferrario et al., 2014; Spalding et al., 2017). However, reef stability is increasingly threatened by anthropogenic stressors, such as climate change, pollution, and sedimentation from land-based runoff (Fabricius, 2005; Vega Thurber et al., 2014; Risk, 2014; Hughes et al., 2017). Sediment carried by runoff from deforestation, agriculture, and urbanization reduces light-availability, smothers coral tissues, and alters reef biogeochemistry (Anthony and Fabricius, 2000; Fabricius, 2005; Junjie et al., 2014; Risk, 2014). Sedimentation further exacerbates other stressors affecting coral health, such as widespread coral bleaching and disease outbreaks, and together these stressors accelerate reef degradation (Tuttle and Donahue, 2022).

Sedimentation affects corals not only through direct physiological stress, but also by altering the surrounding ecological and microbial environment (Fabricius, 2005). Suspended sediments indirectly limit photosynthesis by coral-associated Symbiodiniaceae by decreasing light availability, while direct sediment exposure can impair gas exchange and nutrient uptake by the coral animal itself (Weber et al., 2012; Junjie et al., 2014). Sedimentation also introduces organic matter that fuels microbial metabolism, altering microbial interactions within the coral reef environment (Weber et al., 2012). The severity of these impacts depends on sediment grain size, concentration, and duration of exposure, with fine sediments that remain suspended in the water column longer having the greatest impact on coral reef ecosystems (Fabricius, 2005). While increased sedimentation has been associated with shifts in the microbial community taxonomic composition (Risk, 2014; Tuttle and Donahue, 2022), particularly under chronic conditions, the effects of short-term sedimentation events on coral-associated microbial communities remain relatively unexplored.

The coral surface mucus layer (SML) is a critical interface between corals and their environment, serving as both a physical barrier and niche habitat for diverse microbial communities (Brown and Bythell, 2005; Glasl et al., 2016; Briggs et al., 2021). These microbial assemblages, collectively referred to as the SML microbiome, include bacteria, archaea, fungi, and viruses that contribute to nutrient cycling, pathogen defense, and environmental adaptation (Rohwer et al., 2002; Ritchie, 2006; Bourne et al., 2016; Sweet and Bythell, 2017; Lima et al., 2022). Microbial community composition within the SML microbiome is highly dynamic and shifts in response to fluctuations in water quality, temperature, and coral health (Mouchka et al., 2010; Zou et al., 2022). Stability and diversity in these microbial assemblages are often associated with greater coral health and resilience, however, stress-induced changes can also increase diversity through the introduction of opportunistic or pathogenic taxa, a form of microbial dysbiosis that may signal coral health decline (Kline and Vollmer, 2011; Zaneveld et al., 2016; Hughes et al., 2017; Sweet and Bythell, 2017; Huntley et al., 2022). Some SML-associated microbes play beneficial roles through the production of antimicrobial compounds, but these interactions can be disrupted under environmental stress (Rosenberg and Falkovitz, 2004). For example, thermal stress has been linked to compositional changes and the proliferation of known coral pathogens (Lee et al., 2015), whilenutrient runoff can restructure microbial communities by promoting the growth of bacteria adapted to high-nutrient, low-oxygen conditions (Fabricius, 2005). Sedimentation presents a distinct type of disturbance byphysically smothering coralsand altering the chemical environment of the SML (Weber et al., 2012), favoring heterotrophic bacteria capable of degrading organic matter while suppressing photoautotrophic taxa dependent on light availability (Vega Thurber et al., 2014; Sutherland et al., 2011). Given the central role of microbial interactions in coral health, understanding whether sedimentation-induced changes in microbial diversity and composition are transient or indicative of chronic stress is essential for evaluating coral resilience.

Recent research suggests that prolonged sedimentation can increase microbial diversity in the SML microbiome, often introducing rare or transient microbial taxa during periods of under high sediment loads (Fifer et al., 2022; Miller and Bentlage, 2024). However, the timing and reversibility of these changes remain poorly understood. Specifically, it is unclear how quickly the SML microbiome responds to sediment input and whether short-term sedimentation events lead to similar increases in diversity or community restructuring. Similar taxonomic shifts have been observed under other stressors, such as hypoxia, where elevated abundances of anaerobic bacteria suggest rapid changes in microbial turnover (Howard et al., 2023). Sediment-driven restructuring may similarly favor anaerobic or terrestrial-associated bacteria, potentially displacing host-associated taxa. While some studies have characterized microbial responses to chronic sedimentation, fewer have investigated how acute sedimentation events affect the taxonomic composition and diversity of coral-associated microbiomes or whether such responses are transient or persistent (Fifer et al., 2022). These knowledge gaps limit our ability to evaluate short-term disturbances and predict recovery trajectories in coral microbial communities.

This study examines the effects of sedimentation on the SML microbiome of *Porites lobata* and aims to identify microbial indicators of sediment-induced stress. Specifically, three research questions are addressed: (1) How does sedimentation affect microbiome diversity within the SML of *P. lobata*? (2) How does the taxonomic composition of the SML microbiome change in response to sedimentation? (3) Which bacterial taxa in the *P. lobata* SML microbiome are most responsive to sedimentation events? This study further enhances the understanding of how sedimentation influences coral-associated microbiomes and contributes to the identification of microbial signatures reflective of sedimentation impacts and potential recovery. These insights can help inform the development of microbiome-based tools for monitoring coral reef health and support resilience-based approaches to conservation and management.

## 2 Methods

### 2.1 Study site

The study site, Honoliʻi, Hawaiʻi is a small bay with a narrow beach composed of rounded basalt cobbles to sand size sediment at the base of a sea cliff (Hart, 2018). The beach at Honoliʻi is physically dynamic, shaped by wave action and the input of sediment and freshwater from three streams—Kaiwiki, Maʻili, and Honoliʻi—that converge and discharge through the Honoliʻi Stream mouth (Strauch, 2017). Of these, Honoliʻi Stream is the largest and serves as the primary source of freshwater, sediment, and turbidity into Honoliʻi Bay. Annual rainfall in the Honoliʻi watershed ranges from 3 to 7 m and streamflow discharge ranges from 1.5–6.2 m^3^/s based on annual mean estimates (Giambelluca et al., 2013; USGS, 2024). Approximately 275 m offshore from the Honoliʻi Stream mouth, the cobble and sand substrate transitions to an elevated basalt platform sourced from Mauna Kea lava flows of mid-Pleistocene age (Sherrod et al., 2021). This platform supports a coral reef dominated by *Porites lobata*, *Montipora capitata*, *Montipora flabellata*, and *Pocillopora meandrina* based on visual assessments of coral composition (personal observation). The depth of this reef ranges from 2 to 10 m and is regularly exposed to pulses of sediment and turbidity from stream discharge and north-easterly swells (Puniwai et al., 2016). The close proximity of this coral reef to a recurring turbidity plume generated by Honoliʻi Stream makes it an ideal location to examine changes in coral-associated microbial communities before, during, and after acute sedimentation events.

### 2.2 3D reconstruction and colony selection

**Figure 1:**
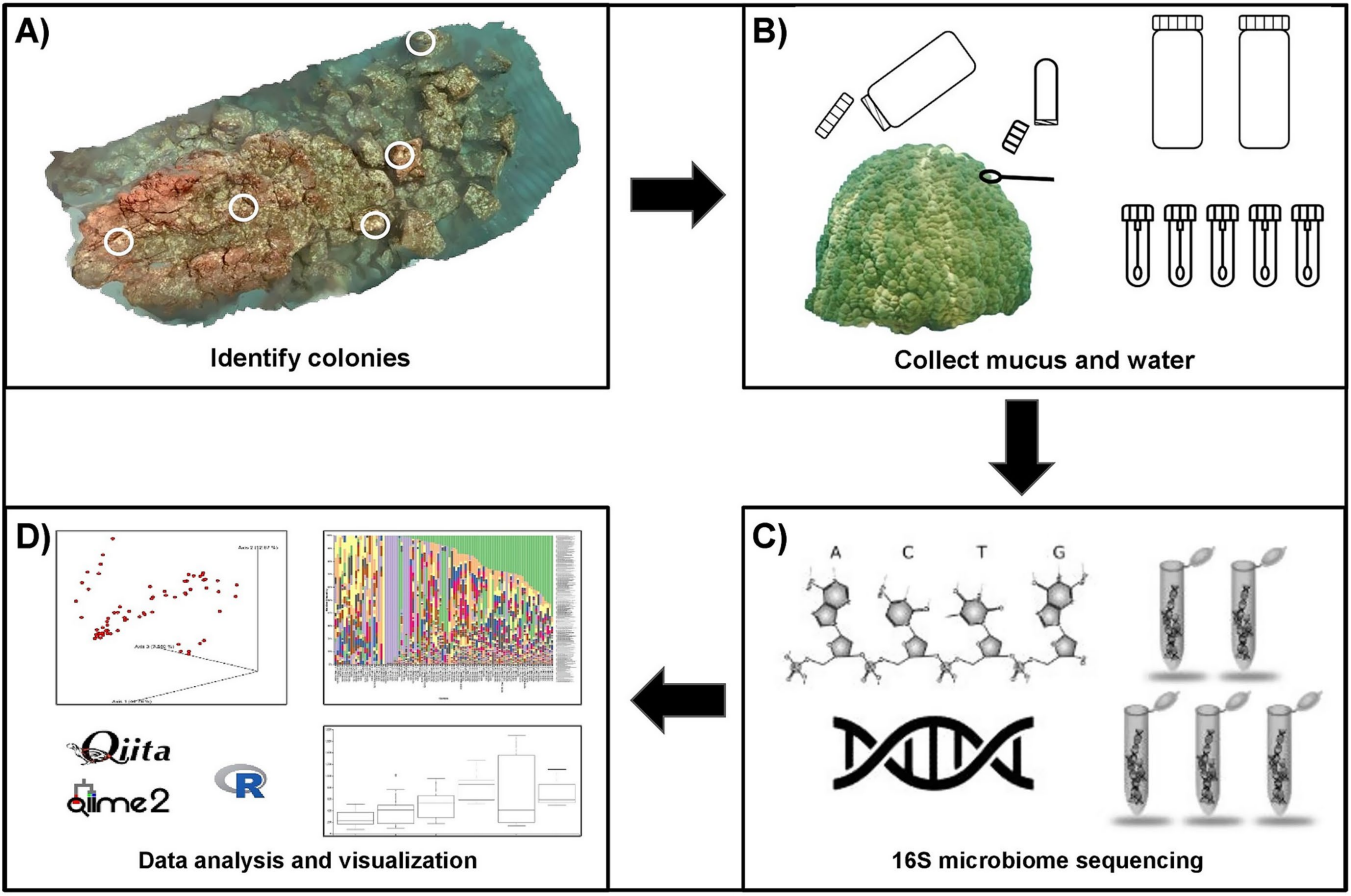
Overview of the study workflow for the characterization of the surface mucus layer (SML) microbiome of *Porites lobata* at Honoli’i, Hawai’i. **(A)** A 3D reconstruction of the reef was created using structure-from-motion photogrammetry. Five *P. lobata* colonies were randomly selected from the 3D reconstruction, with locations confirmed in the field. **(B)** Coral mucus and water were collected before, during, and after a sedimentation event. Two liters of water were collected from the surface and benthos at the center of the reef during each sampling period. Five mucus samples were collected from each colony by divers using SCUBA. For each mucus sample, sterile cotton swabs were stored in microcentrifuge tubes, which were inverted before opening near the coral to retain a sterile air pocket. Swabs were used to gently agitate the coral until mucus was visible, then returned to the sterile tube and transported to the surface by a freediver for storage on dry ice. **(C)** Microbial community profiling was performed by the Knight Lab (UCSD, La Jolla, California) using high-throughput 16S rRNA gene sequencing, targeting the V4 region of the 16S rRNA gene. Amplification was carried out with the primers FWD: GTGYCAGCMGCCGCGGTAA and REV: GGACTACNVGGGTWTCTAAT, following the Earth Microbiome Project (EMP) protocol. Sequencing was conducted on the Illumina MiSeq platform, and taxonomic assignments were made using the Greengenes reference database to the lowest taxonomic level. **(D)** Sequencing data were processed, analyzed, and visualized using Qiita, QIIME2, and R to enable comparative analysis of microbiome composition across samples and sedimentation periods.

Structure-from-Motion (SfM) photogrammetry was used to create a 3D reconstruction of the coral reef using Agisoft Photoscan/Metashape Professional software (Agisoft LLC., St. Petersburg, Russia), following methods adapted from Burns et al. (2015). The reconstruction was used to assess species composition and select colonies for monitoring throughout the sedimentation event. *Porites lobata* was commonly identified across the reef and 10 colonies were randomly selected from the model for potential inclusion in the study. These colonies were then examined in the field for unique morphological features to ensure consistent recognition across sampling periods. Colonies displaying physical signs of stress were excluded. From this selection, five *P. lobata* colonies meeting all criteria were included in the study (Figure 1A).

### 2.3 Sampling periods and collection

**Figure 2:**
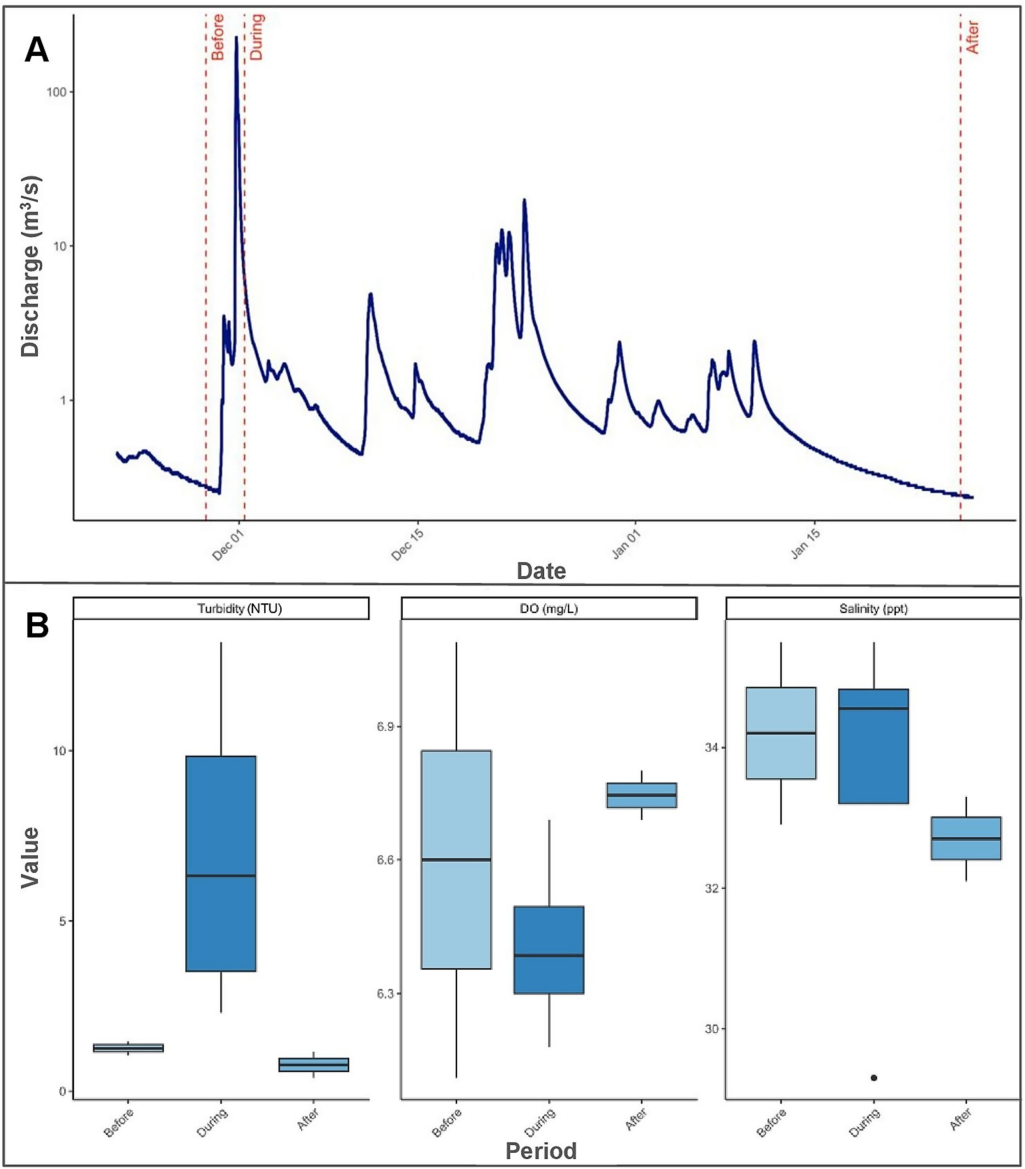
Changes in streamflow discharge and selected water quality indicators across the sedimentation event. **(A)** Time series of Honoliʻi streamflow discharge (m^3^/s) covering the period from 7 days before the sedimentation event to 7 days after the sedimentation event. The discharge is displayed on a logarithmic scale for better visualization and field sampling dates are marked: “Before” (11/28/2023), “During” (12/1/2023), and “After” (1/26/2024). **(B)** Boxplots of turbidity (NTU), dissolved oxygen (DO; mg/L), and salinity (ppt) grouped by sampling period. Each box shows the median (horizontal line) and distribution of values within each period, highlighting differences in water quality before, during, and after the sedimentation event.

Sampling was conducted over a two-month timespan during three distinct sedimentation periods—before, during, and after—a high-streamflow discharge event from Honoliʻi Stream that resulted in a visible sediment plume extending over the study site (Figure 2A). We refer to the physical occurrence of freshwater and sediment input as the sedimentation event, and the associated sampling timepoints as sedimentation periods. Sampling for each sedimentation period was completed in a single day. Before the sedimentation event, sampling took place following a period of low stream discharge, with heavy rainfall expected to occur. Sampling continued when streamflow discharge during heavy rainfall exceeded the 95th percentile for ambient conditions at the Honoliʻi Stream, based on historical data (USGS, 2025). Sampling during the sedimentation event was determined based on real-time monitoring of stream discharge and direct observations of sediment plume extent. The sedimentation event corresponded with a Brown Water Advisory, a public notice issued by the Hawaiʻi Department of Health when runoff may carry pollutants and pathogens into coastal waters, which was in effect from November 30, 2023 to January 4, 2024 (HDOH, 2024). Sampling during the sedimentation event was conducted when environmental conditions, especially underwater visibility, allowed for safe diver operations and accurate colony identification. Sampling after the sedimentation event occurred once ocean conditions, stream discharge, and sedimentation levels returned to pre-event levels. Streamflow discharge was monitored and recorded using real-time data from the U. S. Geological Survey (USGS, 2024) monitoring station at Honoliʻi Stream (Monitoring Location 16,717,000). The daily peak streamflow discharge values reported reflect a period of 7 days before the sedimentation event, 7 days after the first increase in streamflow discharge, and 7 days before the final sampling.

Water and coral mucus were collected during each sedimentation period by a freediver and SCUBA divers deployed from a boat (Figure 1B). Two liters of water (*N* = 2) were collected from the surface and benthic substrate into sterile 1-L polypropylene bottles and stored on ice. Water samples were collected at coral height near on the center of the reef during each period. Five coral mucus samples were collected from each of the five *P. lobata* colonies during each period (*N* = 25). Coral mucus was collected using sterile cotton swabs to gently agitate the coral surface for ~15 s until mucus was visually absorbed. To minimize environmental contamination, swabs were pre-loaded into microcentrifuge tubes and carefully inverted before opening near the coral to retain a sterile air pocket. Once collected, swabs were immediately placed back into the sterile tube, sealed, and transported to the surface by a freediver where they were stored on dry ice until transfer to the lab within 2 h. All mucus samples were inverted during transport to prevent contamination from water intrusion caused by pressure differentials, and any excess liquid was decanted in a sterile environment before storage. Water samples were vacuum-filtered through 0.22-μm mixed cellulose ester membrane filters (Millipore, United States) and transferred to microcentrifuge tubes. All samples were stored at −80°C for later extraction and sequencing.

### 2.4 16S rRNA gene sequencing and quality control

DNA was extracted using the PowerSoil DNA isolation kit (MOBIO Laboratories Inc., Carlsbad, United States). The V4 hypervariable region of the 16S rRNA gene was amplified using polymerase chain reaction (PCR) primers 515F (5′-GTGYCAGCMGCCGCGGTAA- 3′) and 806R (5′-GGACTACNVGGGTWTCTAAT- 3′), following Earth Microbiome Project protocols (Caporaso et al., 2012; 806R, Apprill et al., 2015; 515F, Parada et al., 2016). PCR, library preparation, and sequencing was performed by the Knight Lab (UCSD, La Jolla, California; Figure 1C). Polymerase chain reaction conditions included an initial denaturation at 94°C for 3 min, followed by 35 cycles of 94°C for 45 s, 50°C for 60 s, and 72°C for 90 s, with a final extension at 72°C for 10 min (Caporaso et al., 2023). Dual-indexed libraries were pooled at equimolar concentrations and sequenced on an Illumina MiSeq platform (2 × 300 bp paired-end reads) using sequencing-by-synthesis chemistry. KatheroSeq controls were included contaminate to screen for contamination in low-biomass samples (Minich et al., 2018).

Raw sequence data were processed using Qiita (v2025.02) (Gonzalez et al., 2018), which implements the QIIME2 (v1.9.1) pipeline (Bolyen et al., 2019). Demultiplexing was performed using Split Libraries FASTQ with 12-bp Golay barcodes, allowing a maximum barcode error rate of 1.5%. Adapter and primer trimming was automatically performed using fastp as implemented within the default Qiita workflow.^1^ Sequences were retained at three trimming lengths (90 bp, 100 bp, and 150 bp) to evaluate tradeoffs between taxonomic resolution and sequencing depth. The 100 bp trim length was selected for final analysis, as it provided consistent diversity metrics and retained the highest number of coral mucus samples at the rarefaction threshold.

Closed-reference OTU picking was conducted entirely in Qiita using the QIIME2 (v1.9.1) pipeline with clustering at 97% similarity against the Greengenes reference database (v13_8). Sequences that failed to match the reference database were excluded. Mitochondrial, chloroplast, and eukaryotic sequences were removed using taxonomy-based filtering in Qiita. Only coral mucus samples were retained for downstream analyses, and sequencing blanks and negative controls were excluded during quality filtering.

The final OTU table included 75 coral mucus samples, 10,226 unique OTUs, and 2,636,301 total reads. Median sequencing depth was 29,282 reads per sample (range: 34–88,552). Samples were rarefied to 10,000 reads per sample to normalize sampling effort, based on alpha rarefaction curves for Shannon entropy and observed features that plateaued at this depth (Supplementary Figure S1). The rarefied dataset contained 65 coral mucus samples and 7,786 OTUs, with an even sequencing depth across all samples. A list of sequencing depth per sample is presented in the Supplementary Data S1.

### 2.5 Statistical analyses

All downstream analyses were performed with the rarefied OTU table (100 bp trim length, 10,000 reads/sample) at the family level due to the taxonomic resolution of the data (Figure 1D). Alpha diversity was calculated using Shannon’s entropy and the number of observed features. Statistical differences in alpha diversity were determined with Kruskal-Wallis tests and *post hoc* Dunn’s tests were used to determine significant differences between groups. Beta diversity was calculated using Bray–Curtis dissimilarity and visualized using principal coordinates analysis (PCoA). Group significance was tested using permutational analysis of variance (PERMANOVA) with 999 permutations and pairwise comparisons enabled. Differences in group dispersion were assessed using permutational multivariate analysis of dispersion (PERMDISP), followed by Tukey’s HSD post hoc tests using the vegan (v.2.6–4) package. Alpha and beta diversity analyses were performed in Qiita (v2025.02) and R (v4.3.1).

All remaining analyses were performed in R (v4.3.1) and all visualizations were created using the ggplot2 package (v3.4.4; Wickham, 2016). Relative abundances were calculated by dividing the read count of each taxon by the total number of reads per sample, resulting in proportions that sum to 1 for each sample. These values were multiplied by 100 to express relative abundance as a percentage and summarized by sampling period using the dplyr (v1.1.4; Wickham et al., 2023b) and tidyr (v1.3.1; Wickham et al., 2023a) packages. Only taxa with a mean relative abundance ≥1% in at least one sedimentation period were retained for visualization. Random Forest classification was used to identify taxa that best distinguished between sedimentation periods, using 500 trees in the randomForest package (v4.7-1.1; Liaw and Wiener, 2002) and evaluated with confusion matrices from the caret package (v6.0-94; Kuhn et al., 2023). Feature importance was assessed using Mean Decrease Accuracy (MDA) and Mean Decrease Gini (MDG). Indicator species analysis was performed with the indicspecies package (v1.8.0; Cáceres et al., 2025) using the multipatt function with 999 permutations to identify taxa significantly associated with each sedimentation period.

## 3 Results

### 3.1 Stream discharge and water quality

Streamflow discharge values from the Honoli’i Stream varied greatly throughout the study period (Figure 2A). Peak discharge during the sedimentation event reached 202.75 m^3^/s, with a median of 1.96 m^3^/s (Range [1.78–202.75]), and remained elevated until the final sampling. Median discharge values before and after the sedimentation event were 0.36 m^3^/s [0.28–0.47] and 0.27 m^3^/s [0.24–0.32], respectively. Turbidity over the reef peaked at 10.96 NTU (SD ± 3.17 NTU) during sedimentation, more than triple pre-event (1.26 ± 0.28 NTU) and post-event (0.78 ± 0.54) levels (Figure 2B). Dissolved oxygen decreased from 98.1% (±8.77) before the event to 94.45% (±2.48%) during sedimentation, then increased to 97.50% (±2.12) after the event (Figure 2B). Salinity decreased from 34.2 ppt (±1.84) before the event to 32.4 ppt (±4.38) during the event and slightly increased to 32.7 ppt (±0.85) (Figure 2B). Temperature decreased throughout the event from 25.7°C (±0.28) before to 25.6°C (±0.35) during, and 24.6°C (±0.28) after.

### 3.2 Relative abundance of microbial taxa

**Figure 3:**
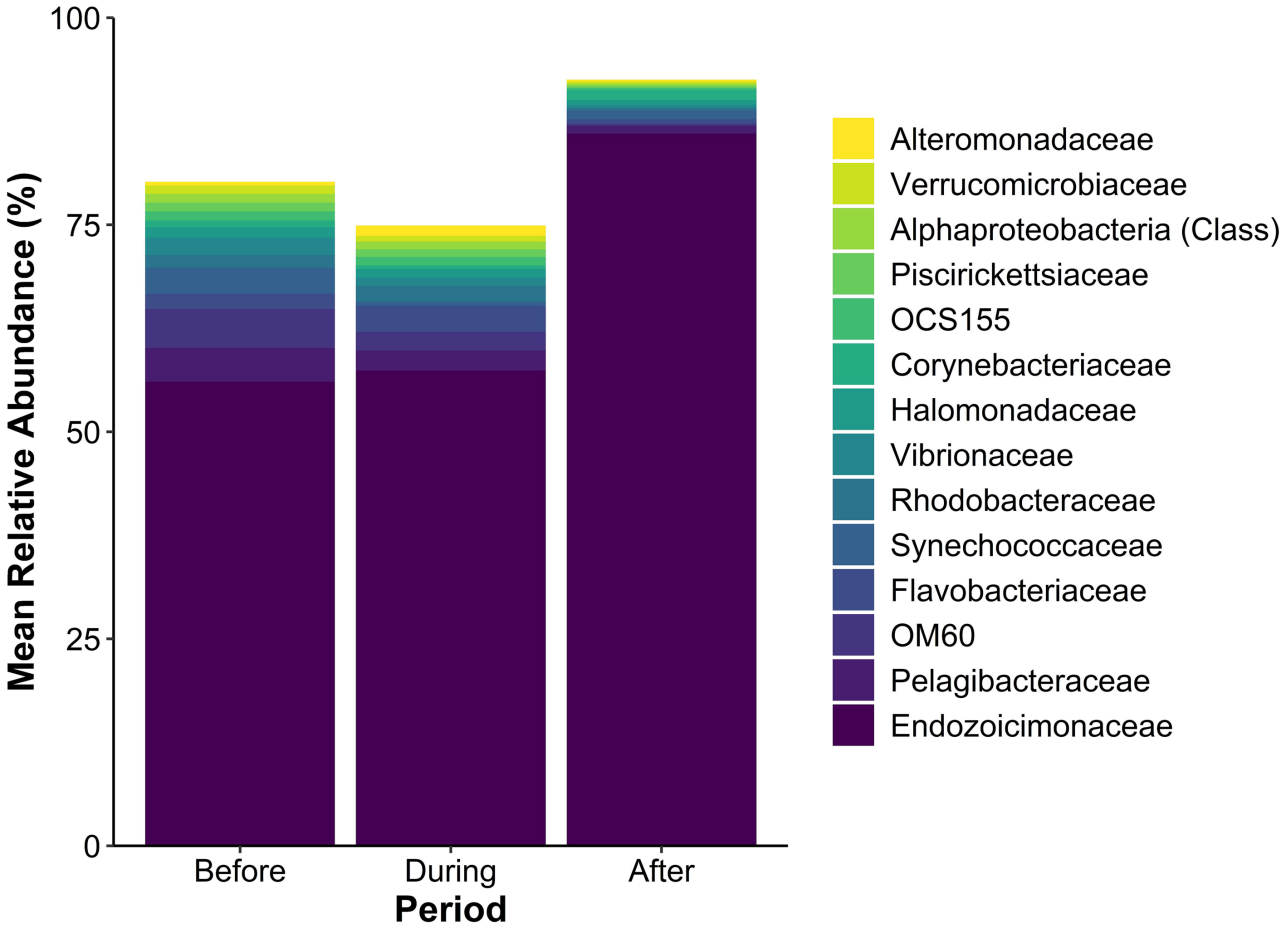
Mean relative abundance (%) of microbial taxa in the SML microbiome of *Porites lobata* coral colonies at Honoli’i, Hawai’i, before, during, and after a sedimentation event. Each bar represents the community composition for each period, with taxa below 1% relative abundance in all periods excluded. Colors indicate specific taxa (see legend), highlighting shifts in community structure associated with the sedimentation event. The figure illustrates changes in microbial dominance and community composition in response to sedimentation. A full list of the mean relative abundance of all taxa present is presented in the Supplementary Data S1.

The SML microbiome of *Porites lobata* exhibited shifts in the community composition across the sedimentation event (Figure 3 shows taxa with a mean relative abundance ≥1% in at least one sedimentation period). The family Endozoicimonaceae dominated all sedimentation periods and increased from 56.0% before, to 57.4% during, and 86.0% after the sedimentation event. The total number of taxa with a mean relative abundance ≥1% before the event was 12, compared to 9 during, and 3 after the sedimentation event. For example, OM60, Pelagibacteraceae, and Flavobacteriaceae each exceeded 3% mean relative abundance before the event, then decreased to less than 1% after the event. Corynebacteriaceae, undetectable before the event, increased to 1.25% mean relative abundance after sedimentation. The relative abundance of all taxa is presented in the Supplementary Data S1.

### 3.3 Alpha and beta diversity

**Figure 4:**
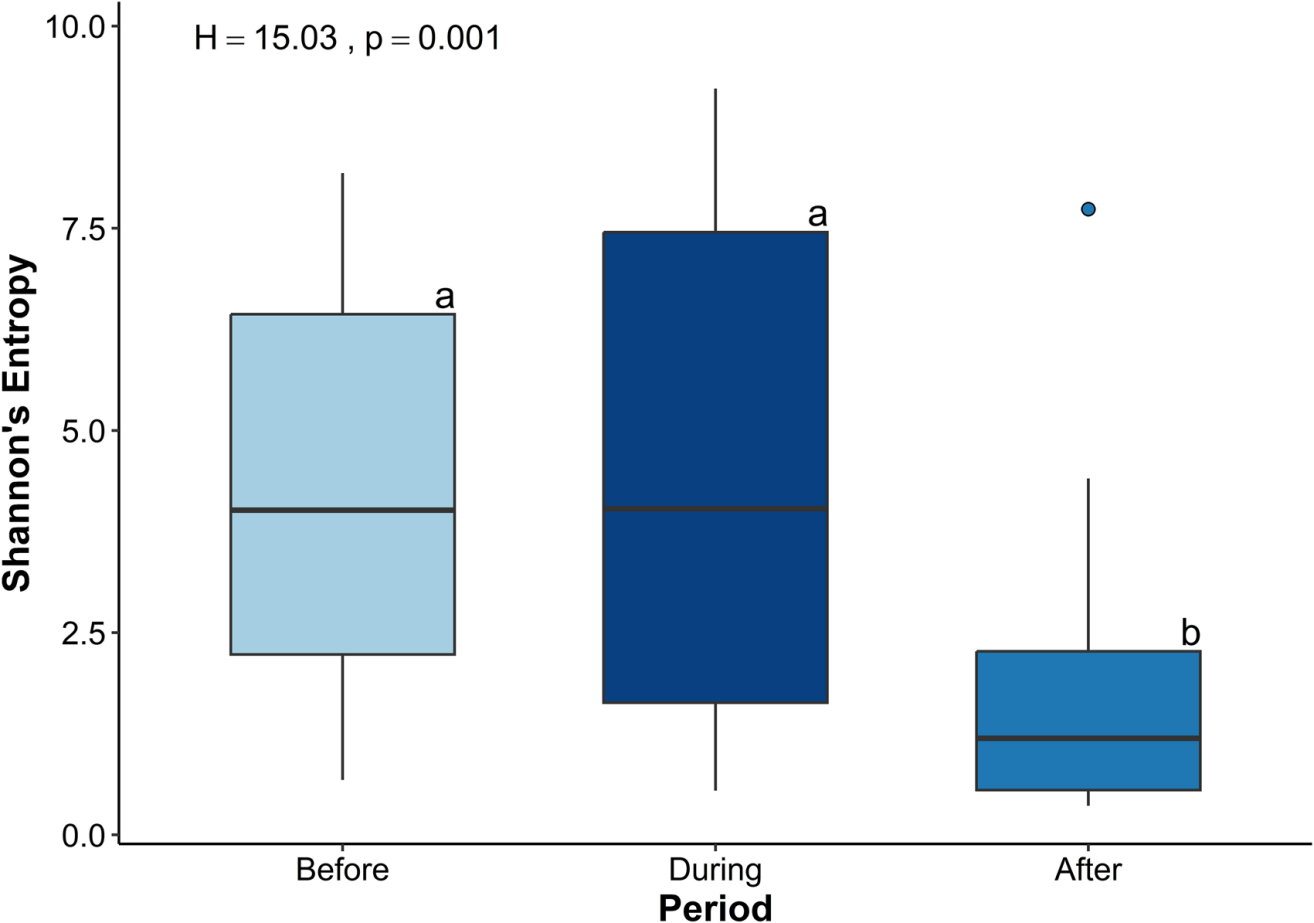
Boxplots of Shannon’s Entropy for microbial communities in the *Porites lobata* surface mucus layer (SML) across three sampling periods before (*N* = 19), during (*N* = 25), and after (*N* = 21) a sedimentation event at Honoli’i, Hawai’i. Shannon’s Entropy (y-axis) is a measure of the microbial diversity that considers abundance and richness of taxa, with higher values indicating greater diversity within the community. Letters (a, b) indicate the statistically significant differences between the time periods determined by a Kruskal-Wallis test (*H* = 15.03, *p* = 0.001). This figure shows a significant decrease in alpha diversity following a sedimentation event.

Shannon’s Entropy varied significantly among the three sampling periods (*H* = 15.03, *p* < 0.001; Figure 4). Shannon’s Entropy was significantly lower after the sedimentation event compared to both during (*H* = 11.61, *p* = 0.001) and before (*H* = 10.83, *p* = 0.001), which were similar (*p* > 0.05). Taxonomic richness, measured as the total number of observed features, also significantly differed among sampling periods (*H* = 30.89, *p* < 0.001; Supplementary Figure S2). Pairwise comparisons showed that taxonomic richness was significantly lower after sedimentation compared to both during (*H* = 24.73, *p* < 0.001) and before (*H* = 13.87, *p* < 0.001) sedimentation, while richness was significantly higher during than before sedimentation (*H* = 7.95, *p* = 0.005; Supplementary Figure S2).

**Figure 5:**
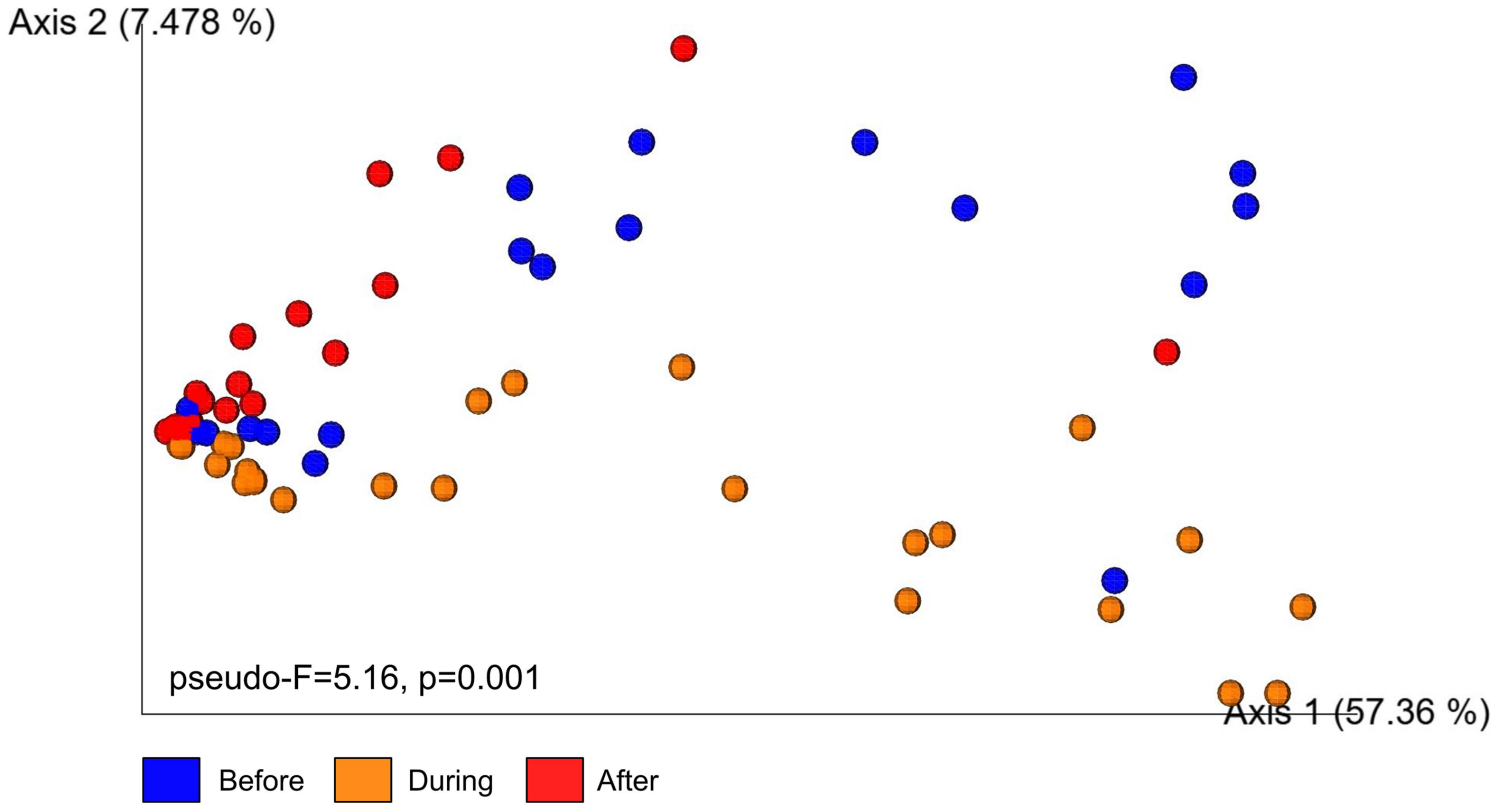
Principal Coordinates Analysis (PCoA) plot illustrates beta diversity of microbial communities in the surface mucus layer of (SML) *Porites lobata* colonies at Honoli’i, Hawai’i before, during, and after a sedimentation event. Each point represents a single sample, with colors indicating the sampling period: blue (Before), orange (During), and red (After). The PCoA is based on Bray–Curtis dissimilarity, a measure that reflects differences in microbial community compositions between samples. Samples that are closer together have more similar communities and samples further apart have more distinct communities. Results from a PERMANOVA analysis (pseudo-*F* = 5.16, *p* = 0.001, 999 permutations, *n* = 65) indicate statistically significant differences in community composition among time periods.

Microbial communities before and during sedimentation formed partially overlapping clusters, indicating some similarity in composition (Figure 5). In contrast, microbial communities after sedimentation formed a distinct and slightly isolated cluster along the secondary axis of variation. A PERMANOVA analysis confirmed significant differences in beta diversity among sampling periods (pseudo-*F* = 5.16, *p* = 0.001, 999 permutations, *n* = 65). Pairwise comparisons identified that beta diversity after sedimentation was different from beta diversity before (*p* = 0.002) and during (*p* = 0.001) sedimentation. Dispersion analysis further showed that microbial communities after sedimentation were significantly less dispersed around their centroid compared to before and during sedimentation (*F* = 6.51, *p* = 0.003; Supplementary Figure S3).

### 3.4 Key taxa from Random Forest analysis

Random Forest classification identified microbial families that best distinguished between sedimentation periods based on family-level relative abundance data. The model was trained using 500 trees and achieved an out-of-bag (OOB) error rate of 12.31%. The overall accuracy of the model was 87.69% (95% ci: 77.18–94.53%), showing strong agreement between predicted and actual sedimentation periods with a Kappa value of 0.8147. Sedimentation period-specific balanced accuracy was highest for the “During” period (0.96), followed by “Before” (0.89), then “After” (0.87). Sensitivity and specificity values exceeded 0.84 across all sedimentation periods.

**Figure 6:**
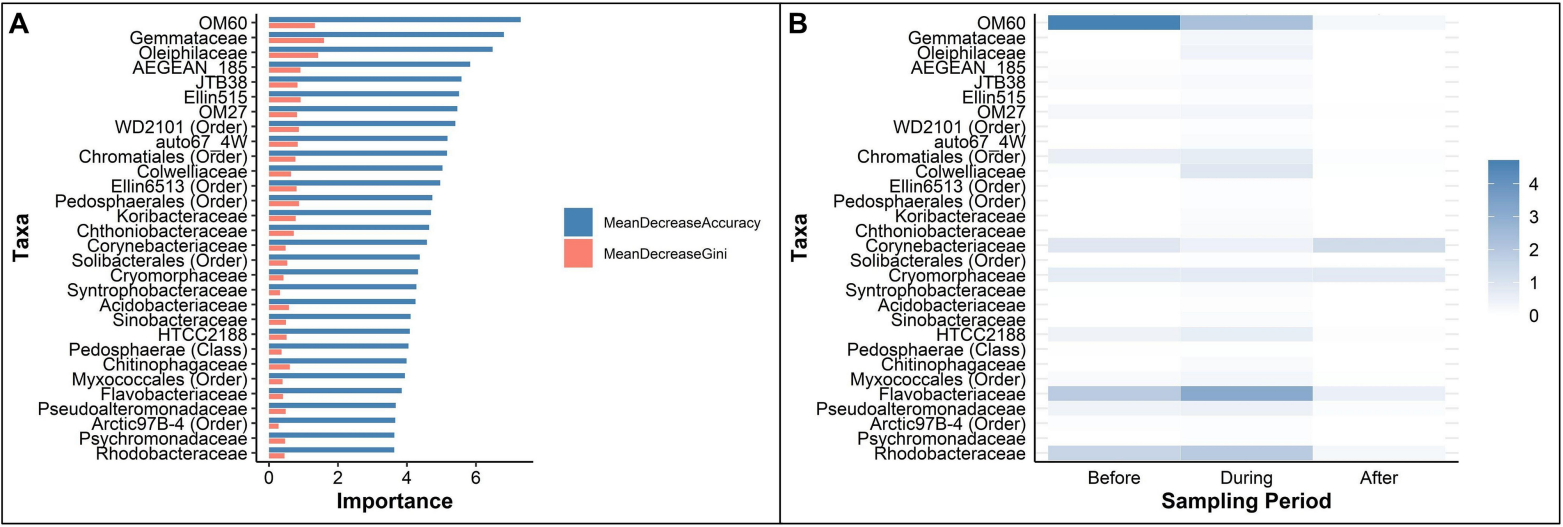
Key taxa, determined by Random Forest analyses, that identified as important in classifying periods before, during, and after a sedimentation event at Honoli’i, Hawai’i. **(A)** The top 30 taxa ranked by Mean Decrease Accuracy (blue) and Mean Decrease Gini (red), representing the most critical taxa to maintain model accuracy in classifying the microbial communities of each period. Higher values indicate greater importance for accurate classification. **(B)** The mean abundance of the top 30 important taxa during each period, ordered by Mean Decrease Accuracy. Darker shades indicate a higher relative abundance, highlighting enrichment patterns before, during, and after the sedimentation event. A full list of taxa that were identified as important by Random Forest classification is presented in the Supplementary Data S1.

Mean Decrease Accuracy (MDA) and Mean Decrease Gini (MDG) values were used to assess feature importance. The top five bacterial families determined by MDA were: OM60, Gemmataceae, Oleiphilaceae, AEGEN_185, and JTB38 (Figure 6A, top 30 taxa). The top five bacterial families determined by MDG were: Gemmataceae, Oleiphilaceae, OM60, Ellin515, and AEGEN_185 (Figure 6A, top 30 taxa). The mean relative abundance of the top 30 important taxa ranked by MDA varied across sampling periods (Figure 6B). The relative abundance of OM60 steadily decreased from 4.71% before to 2.26% during then 0.26% after the sedimentation event. The relative abundance of Flavobacteriaceae increased to 3.14% during sedimentation, compared to 1.83% before and 0.55% after sedimentation. The relative abundance of Corynebacteriaceae slightly declined from 0.84% before sedimentation to 0.45% during sedimentation then finally increased to 1.25% after the sedimentation event. A list of all taxa identified as important by Random Forest classification is presented in the Supplementary Data S1.

### 3.5 Key taxa from indicator species analysis

Several bacterial families were significantly associated with the *P. lobata* SML microbiome for each sedimentation period based on changes in relative abundance. Indicator species analysis identified a total of 206 taxa significantly associated with at least one sedimentation period (*p* < 0.05), 999 permutations: 23 uniquely associated with the Before period, 121 with During, 3 with After, 58 with both Before and During, and 1 with both During and After.

**Figure 7:**
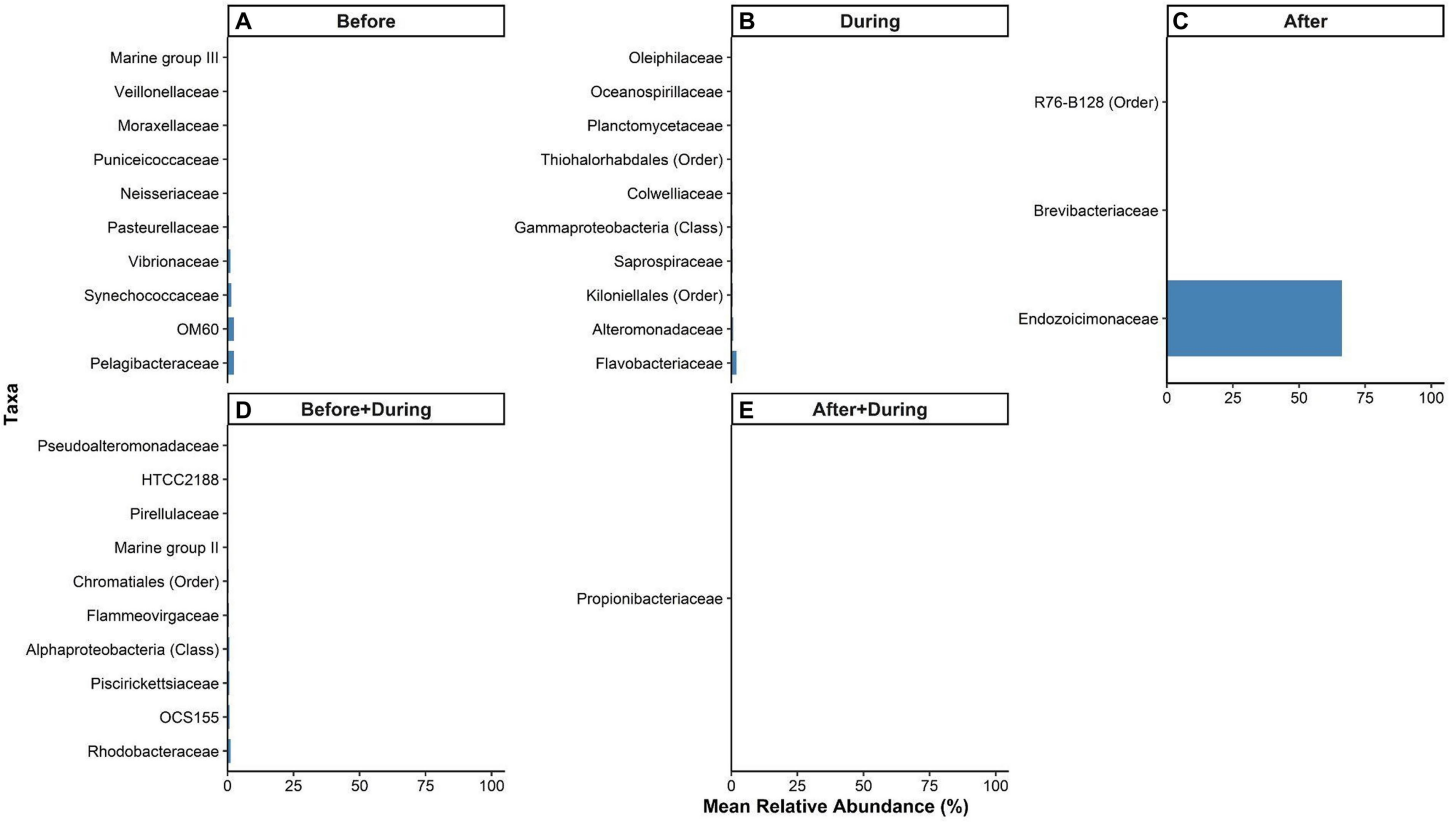
Relative abundances of microbial taxa in the surface mucus layer (SML) of *Porites lobata* significantly associated with each period of a sedimentation event at Honoliʻi, Hawaiʻi (*p* < 0.05). Colored bars represent the relative abundance of taxa associated with the *P. lobata* SML **(A)** before, **(B)** during, and **(C)** after, **(D)** both before and during, and **(E)** both during and after sedimentation. Taxa names are listed in the legend at the lowest identifiable level (Class, Order, or Family). A full list of taxa significantly associated with at least one sedimentation period is presented in the Supplementary Data S1.

The top 10 taxa associated with the *P. lobata* SML microbiome before sedimentation were: Pelagibacteraceae, OM60, Synechococcaceae, Vibrionaceae, Pasteurellaceae, Neisseriaceae, Puniceicoccaceae, Moraxellaceae, Veillonellaceae, and Marine group III (Figure 7A). The top 10 taxa identified as indicators of the microbiome during sedimentation were: Flavobacteriaceae, Alteromonadaceae, an unidentified family in the order Kiloniellales, Saprospiraceae, an unidentified family in the class Gammaproteobacteria, Colwelliaceae, an unassigned family in the order Thiohalorhabdales, Planctomycetaceae, Oceanospirillaceae, and Oleiphilaceae (Figure 7B). The only three taxa identified as indicators of the microbiome after sedimentation were: Endozoicimonaceae, Brevibacteriaceae, and an unassigned family in the order R76-B128 (Figure 7C).

The top 10 taxa identified as indicators of both the before and during sedimentation were: Rhodobacteraceae, OCS155, Piscirickettsiaceae, a family unassigned below the class Alphaproteobacteria, Flammeovirgaceae, a family unassigned below the order Chromataiales, Marine group II, Pirellulaceae, HTCC2188, and Pseudoalteromonadaceae (Figure 7D). The only taxa identified as an indicator of the microbiome both during and after sedimentation was Propionibacteriaceae (Figure 7E). A full list of significant indicator taxa is presented in the Supplementary Data S1.

## 4 Discussion

This study demonstrates that acute sedimentation induced measurable changes in the SML microbiome of *P. lobata* coral, based on observations from five monitored colonies during a documented sedimentation event (HDOH, 2024). Alpha and beta diversity metrics were compared across three sedimentation periods to demonstrate both immediate and lasting sedimentation-driven shifts in the SML microbiome, with the most significant changes observed after sediment exposure. The significant decline in alpha diversity after sedimentation aligns with previous findings that environmental stressors reduce microbial evenness and taxonomic richness (Maher et al., 2020; Bui et al., 2024). Beta diversity analysis further showed that microbial communities were compositionally distinct among sedimentation periods, with the post-event microbiome showing the most divergence from pre- and during-event communities. The patterns of decreased diversity and compositional shifts are consistent with experimental studies of corals exposed to different stressors, such as antibiotics, disease, and hypoxia, suggesting that sedimentation not only decreases microbial diversity, but also restructures community composition (Bent et al., 2021; Guo et al., 2022; Huntley et al., 2022; Howard et al., 2023). These findings demonstrate that acute sedimentation events can drive measurable shifts in both the richness and structure of the *P. lobata* SML microbiome.

Indicator species analyses revealed distinct microbial signatures across sedimentation periods. Before sedimentation, the presence of the coral-associated bacterial families OM60, Synechococcaceae, and Vibrionaceae suggests that a stable, host-associated microbiome is present in the absence of environmental disturbances (McKew et al., 2016; Webster et al., 2016; Greff et al., 2017). During sedimentation 121 families were identified as indicator taxa, reflecting the influx of environmental microbes during sedimentation events. Although commonly associated with coral microbiomes, Flavobacteriaceae was enriched during sedimentation, aligning with prior studies that have observed an enrichment of this group in stressed and diseased corals (Miller and Richardson, 2011; Meyer et al., 2019; Delgadillo-Ordoñez et al., 2022).

Many taxa associated with sedimentation were not retained after the event, suggesting that these microbes may only have a brief influence on the coral SML but do not persist in shaping the microbiome over time. Only three indicator taxa were identified after sedimentation—Endozoicimonaceae, Brevibacteriaceae, and an unassigned family in the order R76-B128—indicating that a more selective community may persist post-disturbance. Among these, Endozoicimonaceae are well-known coral-associated symbionts involved in nutrient cycling, immune modulation, and antimicrobial activity (Neave et al., 2016; Bourne et al., 2016). For instance, *Endozoicomonas marisrubi* has been shown to contribute to the exchange of essential metabolites that could play a role in coral recovery following disturbances (Pogoreutz et al., 2022). Endozoicimonaceae also comprised a large portion of the microbial community during all periods, but was most dominant after sedimentation, suggesting a return to a core microbiome during post-sediment microbiome stabilization (Bernasconi et al., 2019). Brevibacteriaceae and taxa within the R76-B128 order are commonly found in sediments, especially in saline environments, indicating the incorporation of sediment-associated taxa into the SML microbiome post-sedimentation (Forquin-Gomez et al., 2014; van Vliet et al., 2019). The low relative abundance of both Brevibacteriaceae and the unassigned family in the order R76-B128 after sedimentation may be reflective of transient infiltration rather than long-term colonization. Together, these results indicate that while sedimentation can introduce novel microbial taxa to the coral SML, only some are retained beyond the disturbance period. The post-event community appears to reflect both residual sediment-associated taxa and a resurgence of core coral symbionts, highlighting a possible early recovery stage where coral-associated microbes begin to reestablish dominance. The observed trends in microbial community composition across sedimentation periods suggest that microbiome restructuring is a delayed response rather than an immediate shift. Alpha diversity analyses showed that taxonomic richness temporarily increased during sedimentation but significantly declined once environmental conditions returned to pre-event levels. This increase in transient microbe diversity mirrors patterns observed in corals exposed to prolonged sedimentation, where microbial turnover continues beyond the disturbance event itself (Ziegler et al., 2016). Although the subsequent decline in diversity may indicate the onset of recovery, the post-sedimentation microbiome remained compositionally distinct and did not revert to its pre-event state. Moreover, indicator species analysis identified 58 taxa shared between the periods before and during sedimentation and only one taxon shared between the during and after periods, while no taxa were shared between the before and after periods. This progression supports a gradual transition in microbial composition, where few microbial lineages introduced during sedimentation persisted into the recovery period.

While these findings provide new insights into the SML microbiome’s response to an acute sedimentation event, several aspects warrant further investigation. This study focused on a single site, which provided a controlled environmental context, but microbiome composition has been observed to vary over small geographic scales (Wainwright et al., 2019), highlighting the need for multi-site comparisons. Additionally, while 16S rRNA gene sequencing allowed for taxonomic classification, the functional traits and physiological roles of identified taxa remain inferred rather than directly measured. Metagenomic and transcriptomic approaches have proven useful in other studies for identifying microbial genes involved in nutrient cycling, immune modulation, and stress tolerance and could better validate the ecological roles of the taxa highlighted here (Lima et al., 2022; Pogoreutz et al., 2022). The temporal resolution of this study, limited tothe single post-sedimentation time point, provides a snapshot of the SML microbiome nearly 2 months after the sedimentation event and makes it difficult to determine whether the observed composition reflects a temporary disturbance, an intermediate recovery phase, or a longer-term shift in microbiome state. Acute sedimentation events, like the event monitored in this study, are thought to be key drivers of microbiome structuring, but longitudinal sampling is needed to determine whether the microbiome stabilizes over longer periods of time or remains in an altered state (Fifer et al., 2022). Furthermore, microbial shifts may reflect not only host selection, but also changes in the surrounding microbial pool following environmental disturbance. For example, persistent restructuring of microbial communities has been observed in human skin microbiomes after environmental exposures such as water contact and urbanization (Callewart et al., 2020), and similar dynamics may apply to the coral SML microbiome following sedimentation. These changes may represent not only adaptive responses but also the passive acquisition of environmental taxa. Consequently, physiological impacts on the coral host may be influenced as much by environmental disturbance and microbial availability as by selective recovery processes. Despite these considerations, this study provides foundational insights into the taxonomic shifts associated with acute sedimentation and identifies key bacterial families that may serve as indicators of sedimentation stress.

Building on the single-site design of this study, future research should expand to multiple reef locations and incorporate functional analyses to better understand the ecological significance of sedimentation-induced microbiome shifts. Future studies using metagenomic and transcriptomic tools could determine whether observed taxonomic shifts correspond to functional changes, such as antimicrobial compound production or nutrient processing, that directly influence coral health and resilience (Wright et al., 2019; Glaze et al., 2022). Notably, this study revealed short-term loss of diversity, the emergence of sediment-associated and opportunistic taxa during sedimentation, and the retention of potential recovery-associated bacterial families, such as Endozoicimonaceae, after sedimentation. Although the functional roles of taxa associated with the post-sediment microbiome, their identification could help to identify microbial patterns associated with sedimentation stress. These findings highlight the value of identifying sediment-responsive microbial taxa as a first step toward understanding how environmental disturbances shape coral microbiomes, and they underscore the importance of linking taxonomic shifts with both microbial function and coral health outcomes in future studies.

Microbial responses to sedimentation are likely to be habitat-specific, thus future studies should include multiple reef sites impacted by acute sedimentation to determine whether consistent patterns in microbial shifts emerge across locations (Glasl et al., 2019). Extended time series from multiple seasons and recovery stages will help differentiate natural variability from disturbance-driven shifts, particularly given the failure of the microbiome to return to pre-event composition nearly 2 months after the initial sediment exposure and seasonal variability reported in corals elsewhere (Miller and Bentlage, 2024). Controlled sediment exposure experiments could further establish causal links between sedimentation and microbial community restructuring. For example, the persistence and increase in taxa such as *Endozoicimonaceae* warrants further investigation into its potential role in post-sedimentation microbial community dynamics and recovery processes (Pogoreutz et al., 2022). These foundational insights are a necessary precursor to the development of microbiome-based restoration strategies, such as probiotics or microbial transplants, to mitigate sedimentation stress and enhance coral resilience (Moradi et al., 2023; Delgadillo-Ordoñez et al., 2024).

Understanding how sedimentation-driven microbiome shifts relate to overall coral resilience is essential for predicting coral responses to increasing sedimentation pressure. If microbial restructuring following sedimentation disrupts key microbial functions, such as nutrient cycling, pathogen defense, or symbiosis, corals may become more vulnerable to additional stressors, including warming, pollution, or disease outbreaks (Vezzulli et al., 2013; Neave et al., 2016; Webster et al., 2016). However, it remains unclear whether these shifts represent a temporary stress response or a functional adaptation that enhances coral resilience. Clarifying the ecological roles of sediment-responsive taxa is therefore critical for developing microbiome-based conservation strategies (Boilard et al., 2020; Terzin et al., 2024). Building on this study’s findings, future research should also consider how microbiome assembly is influenced by environmentally available microbes, recognizing that community shifts may be driven as much by stochastic colonization as by host filtering. As land-based runoff continues to threaten nearshore coral reefs, these insights underscore the importance of integrating microbiome dynamics into coral conservation frameworks and provide a foundation for identifying microbial signatures of sedimentation stress and recovery (Staley et al., 2017; Carlson et al., 2019).

## Data Availability

Processed microbial data used in this study, including sample metadata, taxonomic assignments, and OTU tables, are provided as Supplementary Data S1. Raw sequencing data generated for this study have been deposited in the European Nucleotide Archive (ENA) database under project accession number PRJEB91376 (https://www.ebi.ac.uk/ena/browser/view/PRJEB91376).

## Footnotes

^1^ https://qiita.ucsd.edu/static/doc/html/processingdata/qp-fastp-minimap2.html?highlight=adapter

## Supplementary material

Supplementary Data S1 See the README tab in the file.

Supplementary Figure S1 Alpha rarefaction curves showing Shannon’s entropy for microbial communities in the *Porites lobata* surface mucus layer (SML) across three sedimentation periods. Curves were generated across a range of sequencing depths (x-axis) to evaluate whether diversity estimates stabilized.

Supplementary Figure S2 Boxplots showing the number of observed features in coral mucus microbiomes before, during, and after a sedimentation event at Honoliʻi, Hawaiʻi. Letters indicate statistically significant differences between groups based on a Kruskal-Wallis test (*H* = 30.88, *p* < 0.001) with lower case letters indicating significant differences between groups.

Supplementary Figure S3 Boxplot of microbial community dispersion across three sampling periods before (*N* = 19), during (*N* = 25), and after (*N* = 21) a sedimentation event at Honoli’i, Hawai’i, based on distance to centroid values from a PERMDISP analysis (*F* = 6.51, *p* = 0.003). Distance to centroid (y-axis) represents the variability in microbial community composition within each group, with lower values indicating more homogenous communities. Lower case letters indicate significant differences between groups.
