# Supplementary figures and images for: Sediment exposure decreases diversity in the surface mucus layer microbiome of Porites lobata at Honoliʻi, Hawaiʻi
Source: Front Microbiol. 2025 Jul 28;16:1626064. doi: 10.3389/fmicb.2025.1626064 (PMC12336141; doi:10.3389/fmicb.2025.1626064)

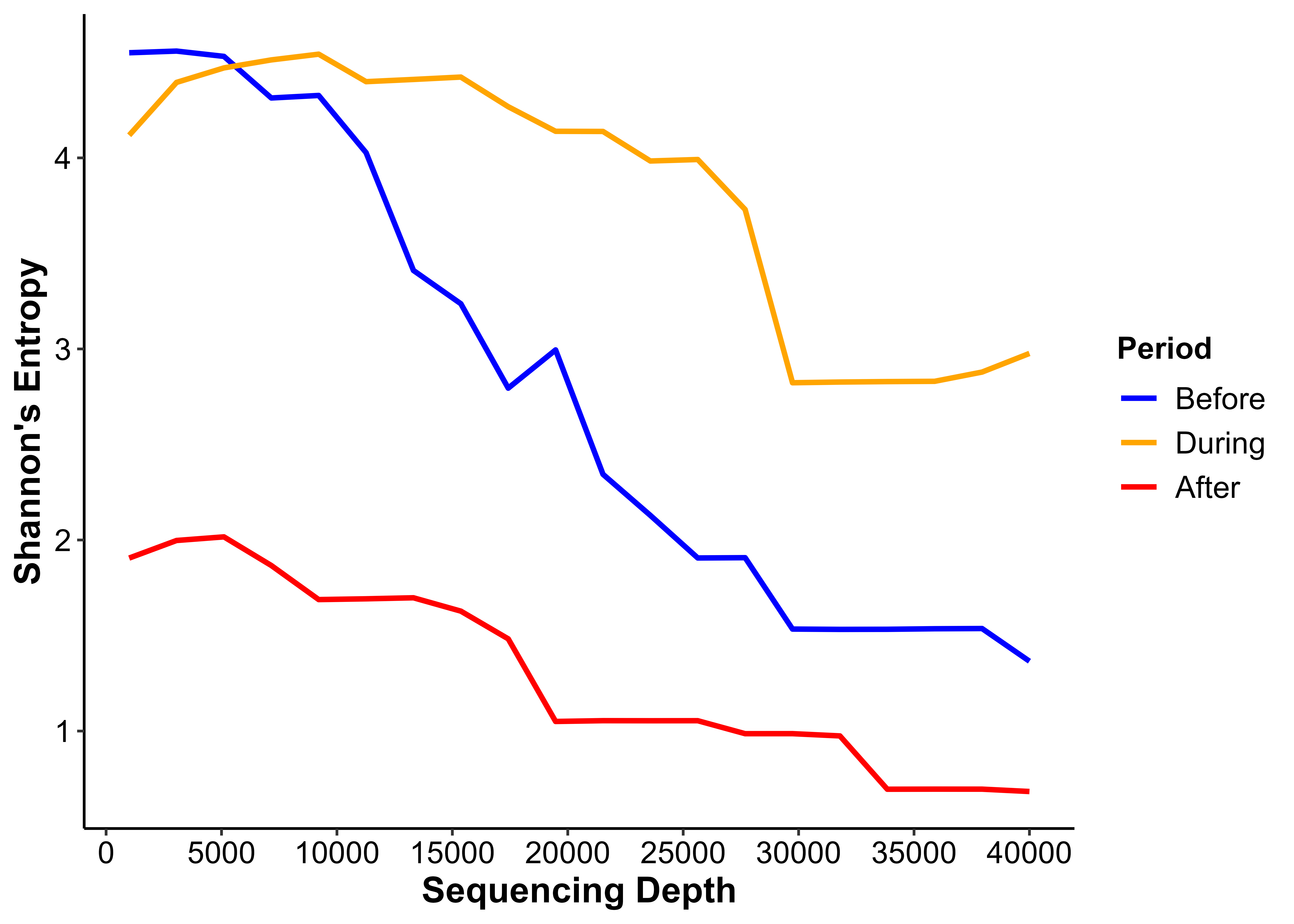

Supplement: Supplementary Figure S1 — Alpha rarefaction curves showing Shannon’s entropy for microbial communities in the Porites lobata surface mucus layer (SML) across three sedimentation periods. Curves were generated across a range of sequencing depths (x-axis) to evaluate whether diversity estimates stabilized. [file Image_1.jpeg]

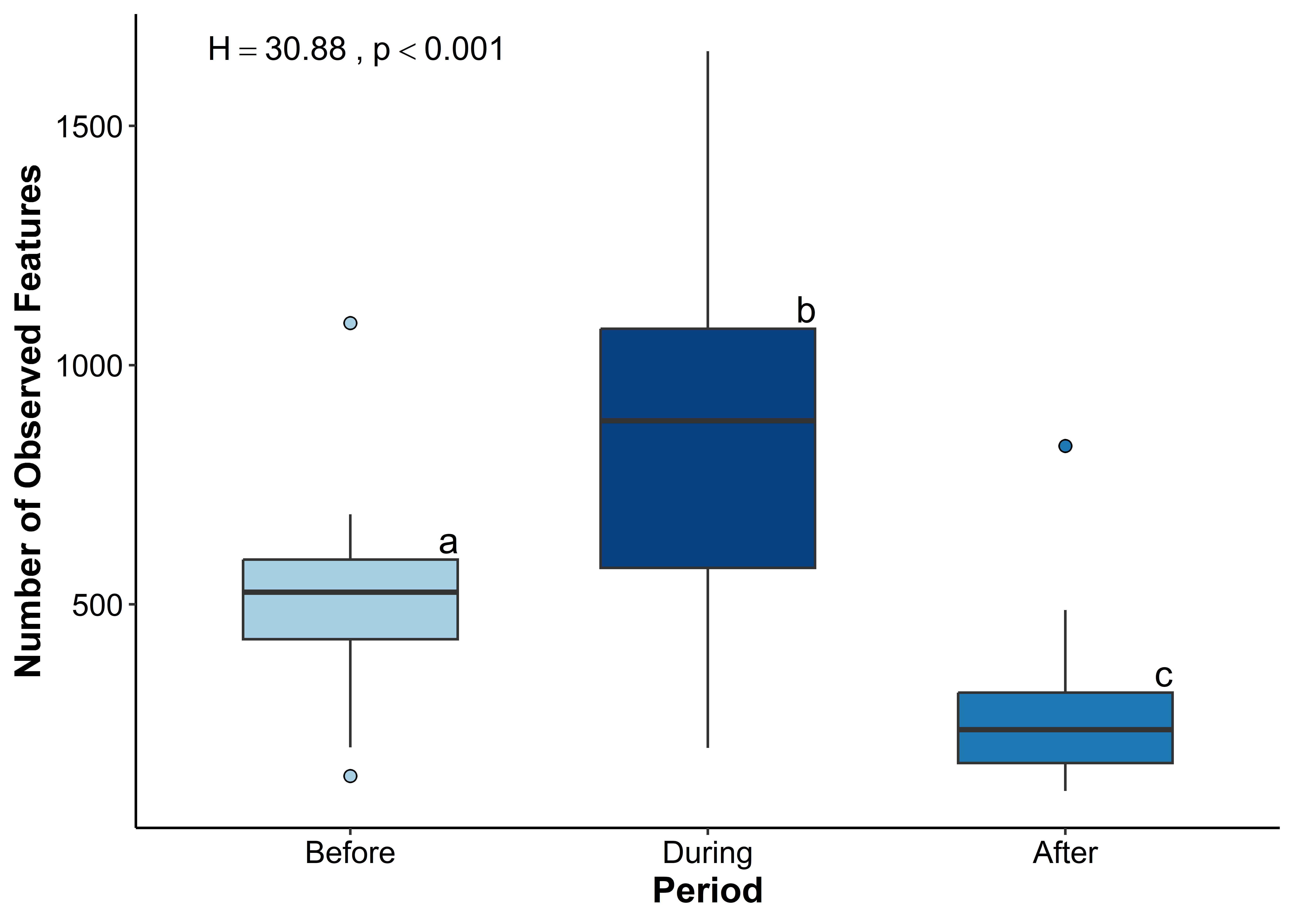

Supplement: Supplementary Figure S2 — Boxplots showing the number of observed features in coral mucus microbiomes before, during, and after a sedimentation event at Honoliʻi, Hawaiʻi. Letters indicate statistically significant differences between groups based on a Kruskal-Wallis test (H = 30.88, p < 0.001) with lower case letters indicating significant differences between groups. [file Image_2.jpeg]

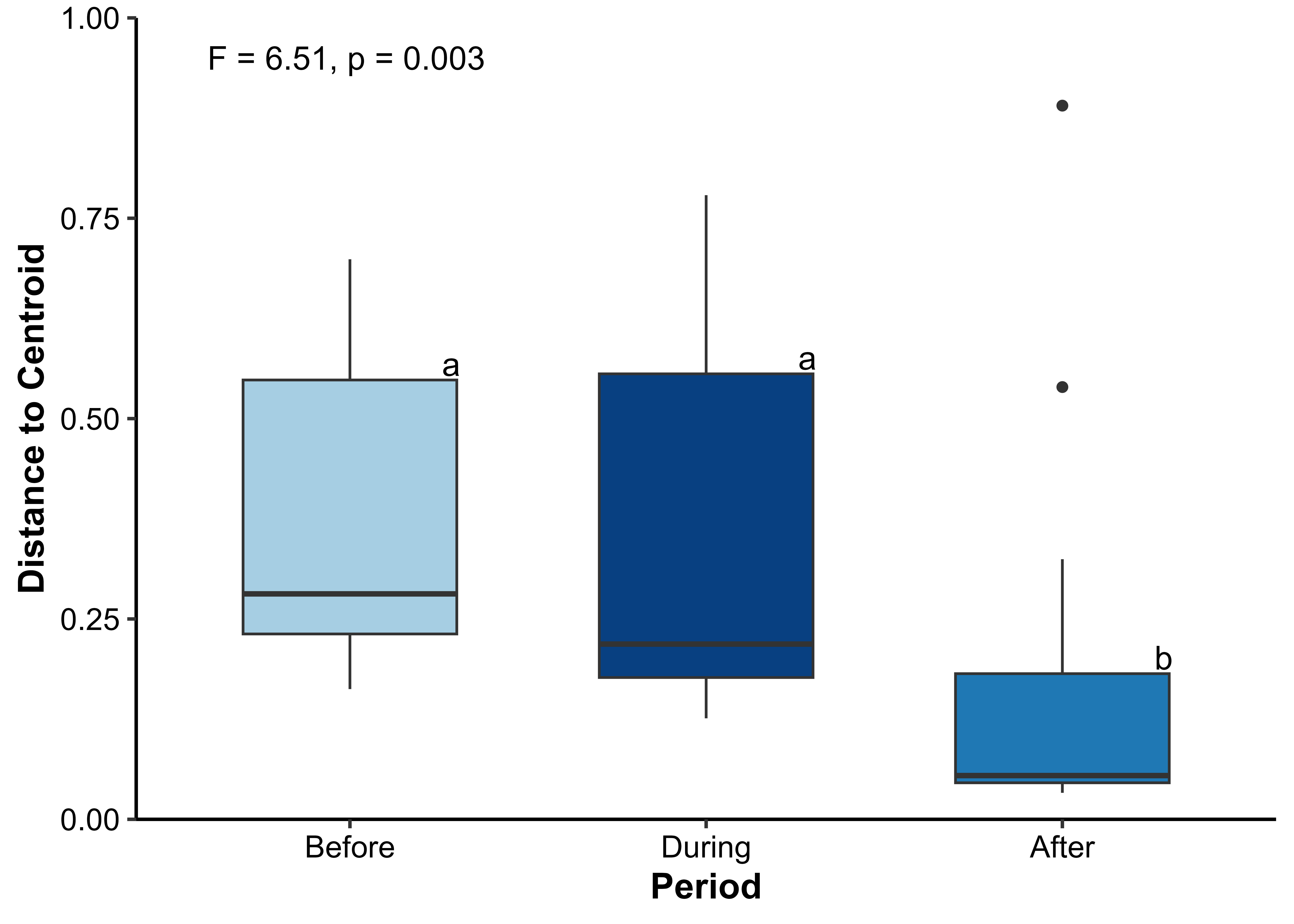

Supplement: Supplementary Figure S3 — Boxplot of microbial community dispersion across three sampling periods before (N = 19), during (N = 25), and after (N = 21) a sedimentation event at Honoli’i, Hawai’i, based on distance to centroid values from a PERMDISP analysis (F = 6.51, p = 0.003). Distance to centroid (y-axis) represents the variability in microbial community composition within each group, with lower values indicating more homogenous communities. Lower case letters indicate significant differences between groups. [file Image_3.jpeg]
